# Insights from a 7-Year Dementia Cohort (VALCODIS): ApoE Genotype Evaluation

**DOI:** 10.3390/jcm13164735

**Published:** 2024-08-12

**Authors:** Miguel Baquero, Laura Ferré-González, Lourdes Álvarez-Sánchez, Inés Ferrer-Cairols, Lorena García-Vallés, Mar Peretó, Luis Raga, Gemma García-Lluch, Carmen Peña-Bautista, Beatriz Muria, Aitana Prieto, Inés Jareño, Consuelo Cháfer-Pericás

**Affiliations:** 1 Research Group in Alzheimer’s Disease, Instituto de Investigación Sanitaria La Fe, Avda. Fernando Abril Martorell, 106, 46026 Valencia, Spain; baquero_miq@gva.es (M.B.); laufegon@alumni.uv.es (L.F.-G.); lourdes_alvarez@iislafe.es (L.Á.-S.); ines_ferrer@iislafe.es (I.F.-C.); lorena_garcia@iislafe.es (L.G.-V.); mar_pereto@iislafe.es (M.P.); luis_raga@iislafe.es (L.R.); gemma.garcia2@alumnos.uchceu.es (G.G.-L.); mariadelcarmen_pena@iislafe.es (C.P.-B.); beatriz_muria@iislafe.es (B.M.); aitana_prieto@iislafe.es (A.P.); ines_jarenyo@iislafe.es (I.J.); 2 Neurology Unit, University and Polytechnic Hospital La Fe, Avda. Fernando Abril Martorell, 106, 46026 Valencia, Spain

**Keywords:** cognitive disease, dementia, Alzheimer’s, biomarkers, neuropsychology, ApoE

## Abstract

**Background:** The VALCODIS (Valencian Cognitive Diseases Study) cohort was designed and studied at the Hospital Universitari i Politècnic La Fe (Valencia, Spain) for the research of cognitive diseases, especially in the search for new biomarkers of Alzheimer’s disease (AD). **Methods:** Participants in the VALCODIS cohort had cerebrospinal fluid (CSF) and blood samples, neuroimaging, and neuropsychological tests. The ApoE genotype was evaluated to identify its relationship with CSF biomarkers and neuropsychological tests in AD and non-AD participants. **Results:** A total of 1249 participants were included. They were mainly AD patients (*n* = 547) but also patients with other dementias (frontotemporal lobar dementia (*n* = 61), Lewy body dementia without AD CSF signature (*n* = 10), vascular dementia (*n* = 24) and other specific causes of cognitive impairment (*n* = 442), and patients with subjective memory complaints (*n* = 165)). In the ApoE genotype evaluation, significant differences were found for Aβ42 levels between genotypes in both AD and non-AD patients, as well as a negative correlation between tau values and a cognitive test in non-carriers and ε4 heterozygous. **Conclusions:** The VALCODIS cohort provides biologically diagnosed patients with demographical, clinical and biochemical data, and biological samples for further studies on early AD diagnosis. Also, the ApoE genotype evaluation showed correlations between CSF biomarkers and neuropsychological tests.

## 1. Introduction

In recent years, there has been an increase in the number of people suffering from dementia, a fact that is surely due to the increase in life expectancy [1,2]. The number of people affected is expected to increase to 150 million by 2050 [3,4,5]. Dementia is defined as an acquired pathology that causes induced disability secondary to impairment and decline in several cognitive domains (memory, language, executive function, attention, perceptual–motor, social cognition, learning capability), severe enough to interfere with daily life [6,7]. Alzheimer’s disease (AD) is the most common cause of dementia (approximately 60–80%). Other non-Alzheimer dementias are dementia with Lewy bodies (DLB), Parkinson’s disease dementia, frontotemporal lobar degeneration (FTLD) [8,9], progressive supranuclear palsy, corticobasal degeneration, multisystem atrophy, (with the last three included in a extended concept of FTD), argyrophilic grain disease, Huntington’s disease and others [10]. Some other diseases that strictly cannot be considered as neurodegenerative can also be a cause of cognitive decline, especially vascular cognitive impairment or vascular dementia (VaD) spectrum [11]. In anticipation of the increase in the prevalence of this pathology, there is a current need to search for new biomarkers to diagnose neurodegenerative diseases, as current methods are based on invasive and expensive techniques, and some of those aetiologies do not have specific markers.

There are different types of risk factors for dementia, including medical, lifestyle, and environmental factors. AD is a multifactorial disease with some risk factors, the Apolipoprotein ε4 (ApoE4) genotype being the strongest genetic risk factor [12,13]. Carrying the ε4 isoform has been associated with earlier amyloid deposition [14], and people with AD had the highest frequency of Apo E4/E4 genotypes [15]. Furthermore, some studies found differences in biomarkers among the ε4 carriers or non ε4-carriers [16]; even in plasma from early AD patients, different metabolic profiles were obtained depending on their ApoE genotype [12].

Many research projects have been developed in recent decades to find more accessible and inexpensive biomarkers with diagnostic and prognostic value. Therefore, following the same line of research and to increase our knowledge of the pathology and the factors associated with it, the VALCODIS (Valencian Cognitive Diseases Study) cohort was designed and examined.

The Cognitive Disorders Unit forms part of the Neurology Department of the Hospital Universitari i Politècnic La Fe (HULAFE), a public national hospital, primarily responsible for medical specialties for approximately 300,000 inhabitants in the region of Valencia (Spain) and a reference tertiary centre for numerous diseases. The Cognitive Disorders Unit of Neurology Department of the HULAFE focuses its attention on three aspects: assistance activity, research and standardisation, and improvements in modern techniques for early diagnosis (neuropsychology, laboratory, and neuroimaging). Therefore, the specialised neurology appointments of this unit attend to patients with complaints or cognitive or behavioural symptoms that have been referred from primary care, directly from the neurology unit or by a number of other ways, including self-reference from sanitary workers at HULAFE.

**Figure 1:**
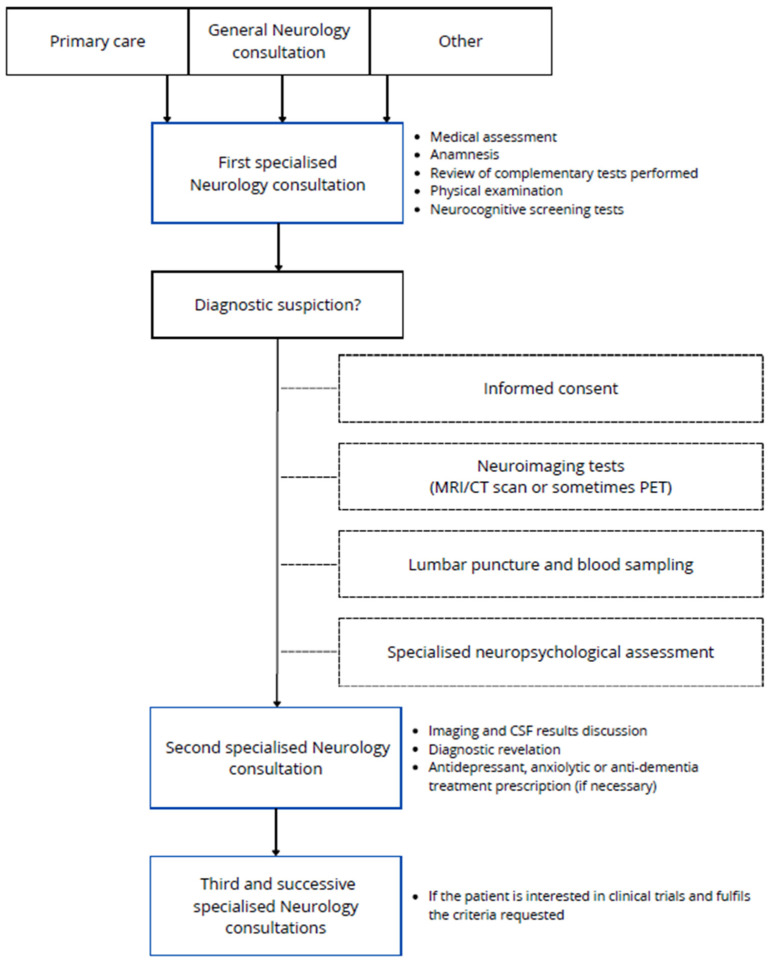
Schematic representation of the data collection process for the VALCODIS cohort.

The participant recruitment process is summarized in Figure 1. The first visit of the patients was with one of the neurologists of the research group in AD, who carried out a medical assessment, anamnesis, physical examination, and commonly performed some neurocognitive screening tests. The patients were mostly referred from primary care but also from general neurology consultation and consultations of other specialities or other ways. If an aetiologic disease diagnosis is suspected, patients will be rescheduled for neuroimaging tests if they are not available (MRI/CT scan or sometimes PET), general blood sampling, amyloid status determination by CSF analysis or amyloid brain PET, and finally specialised predefined neuropsychological assessment, realized in one session of interviews spanning no more than 2.5–3 h. When these other tests are available, a second specialised consultation is held with the neurologists, who re-evaluate the patient and complement data in order to establish the diagnosis, when possible. Rarely, other supplementary tests could be requested. Also, some treatments could be prescribed according to the diagnosis. If the patient is interested in clinical trials and fulfils the requested criteria, participation can be offered to them in a subsequent consultation. Clinical trials are carried out by a concrete group of members of the research team, including neurologists, neuropsychologists, nurse coordinators, and data managers. This team is responsible for reviewing trial protocols to organise future follow-up visits and, when required, drug administration. 

## 2. Methods

### 2.1. VALCODIS Cohort: General Protocol

The VALCODIS cohort was launched in 2017 without interruption with the aim of conducting an observational study to discover and validate new biomarkers of different natures (lipids, proteins, metabolites, microRNAs...) that will help in early and minimally invasive AD diagnosis. Also, it will improve the early initiation of currently available conventional treatments, as well as the possibility of participating in clinical trials. The VALCODIS cohort includes patients diagnosed with AD, DLB, FTLD, VaD, and other cognitive impairment causes (psychiatric, with or without dementia but with negative biomarkers for AD; some patients became unable to be classified or have incomplete data), and subjects with subjective memory complaints (SMCs) without evidence of neurodegenerative disease.

Regarding general inclusion criteria, they are people who speak and/or understand the Spanish language to fully comprehend the study information and cognitive tests, aged between 40 and 80 years, and signed informed consent for lumbar puncture (or alternatively amyloid PET scan), blood sample extraction, neuropsychological tests and neuroimaging. On the other hand, general exclusion criteria included patients who refused to participate, who have significant psychiatric disorders or other comorbidities that would affect cognitive abilities, and patients with advanced disease who cannot undertake the neuropsychological testing. Therefore, patients with severe cognitive impairments due to neurodegenerative diseases were excluded. 

Regarding classification criteria, SMC patients are subjects with negative levels for CSF AD biomarkers and mild cognitive complaints, as assessed by a specialist in neuropsychology, without functional repercussions in their daily living. The mild cognitive impairment (MCI) due to the AD group includes patients with positive CSF AD biomarkers and impairments in at least one cognitive domain, without an impairment in daily function. The mild dementia AD group includes patients with positive CSF AD biomarkers and cognitive impairment in one or more cognitive domains that precludes activities of daily living. The VaD group included negative CSF AD biomarkers and cognitive impairment, as defined by the International Society of Vascular Behavioural and Cognitive Disorders (VAS-COG) criteria [17]. The DLB group included patients with cognitive impairment who meet the criteria developed by the DLB Consortium [18], presenting negative CSF AD biomarkers. This group included the probable DLB (two or more core clinical features of DLB are present, with or without indicative biomarkers, or only one core clinical feature is present but with one or more indicative biomarkers) and possible DLB (only one core clinical feature of DLB is present, with no indicative biomarker evidence, or one or more indicative biomarkers are present, but there are no core clinical features) subgroups. The FTLD group included patients with negative CSF AD biomarkers. The FTD group included patients meeting the International Behavioural Variant FTD Criteria Consortium (FTDC) [19] criteria or the international expert consensus in 2011 for the diagnosis of Primary Progressive Aphasia [20]. These classification criteria used in the VALCODIS study are specified in Table S1 (see Supplementary Material).

### 2.2. Ethical Considerations

The details of the protocol are explained by the clinicians at the initial visit, after which signed informed consent is obtained from all participants, in which they agree to perform a lumbar puncture, blood sampling, storage of biological samples, neuropsychological testing, and neuroimaging. The Ethics Committee (CEIC) at the Health Research Institute La Fe (Valencia, Spain) approved the study protocols (2016/0257 10 October 2016; 2019/0105 12 April 2019; 2022-282-1 8 June 2022; 2016/116; 2021/454-1), and informed consent was obtained from all study participants.

Moreover, the participants in this study are identified by a code, so that no information that could identify any participant in the study is included.

### 2.3. Sociodemographic and Anthropometric Data Collected

Participants’ data including demographic information and life habits were collected. The demographic information included age, sex (masculine/feminine), educational level (basic/primary/secondary/college), profession and employment situation (active/unemployed/retired), hand dominance (right-handed/left-handed), weight (kg), height (cm) and body mass index (BMI). Moreover, life habits, including tobacco use (yes/no, amount), alcohol use (yes/no, amount and type), physical exercise (yes/no, less than 150 min per week/ more than 150 min per week), and information of the accompanying relative, were added (relationship, name).

### 2.4. Clinical, Neuropsychological, Image, and Biomarker Data

#### 2.4.1. Clinical Data

During the first visit with neurologists, data related to the patient’s symptoms (memory, language, behaviour...) were collected. Also, their complete pharmacological profile (statins, fibrates, antidepressants, antiepileptics, anticoagulants, anti-inflammatories…) was registered, including their comorbidities (dyslipidaemia, diabetes, hypertension, heart disease…).

#### 2.4.2. Neuropsychological Evaluation Data

After an initial evaluation with neurologists, all VALCODIS participants are evaluated via a standard two-hour neuropsychological performance. From the outset, the standard protocol includes six scales to assess cognition, daily living functionality, and depression. The battery consists of the following scales: Clinical Dementia Rating (CDR) [21,22], used to characterize six domains of cognitive and functional performance, such as memory, orientation, judgment and problem solving, community affairs, home and hobbies, and personal care, with an interview with the patient and their caregiver; the Mini-mental State Examination (MMSE) [23,24], a screening tool used to assess mental status through five areas of cognition, orientation, registration, attention and calculation, recall, language (naming, repetition, comprehension, reading and writing), and drawing; repeatable battery for the Assessment of Neuropsychological Status (RBANS) [25,26] is a battery, which includes twelve subscales grouped in five domains (immediate memory, visuospatial construction, language, attention and delayed memory); the Mini-Cog-Functional Activities Questionnaire (FAQ) [27] and the Alzheimer’s Disease Cooperative Study–Activities of Daily Living Inventory-Mild Cognitive Impairment (ADCS-ADL-MCI) [28,29] were both used to assess the patient’s ability to perform basic and instrumental daily living activities; and the Geriatric Depression Scale (GDS) [30], a 30-item self-administered questionnaire used to screen for depression symptoms in the elderly and evaluate the clinical severity of depression.

#### 2.4.3. Image Data

Neuroimaging with a head computed tomography (CT) or magnetic resonance imaging (MRI) scan was performed on the patients, following the American Academy of Neurology recommendations to perform structural neuroimaging with non-contrast cranial CT or MRI in the routine initial evaluation of all patients with dementia [31]. The procedure consisted of a 1.5 tesla brain MRI. In most cases, MRI was preferred over CT because it has the best sensitivity for detecting and evaluating potential brain pathologies and avoiding ionising radiation. The non-contrast brain MRI protocol included T1-weighted sequences (to see anatomy), protonic density/T2/FLAIR (to evaluate the presence of oedema or other abnormalities), gradient echo sequence (to evaluate the presence of micro/microhaemorrhages), and a diffusion sequence (to see the presence of acute alterations). The most relevant structural imaging findings included cerebral atrophy (generalised or regionally localised) [32], ventriculomegaly, ischemic cerebrovascular disease (strokes and diffuse white matter ischemic changes), and microhaemorrhages (observed in vascular anomalies, cerebral amyloid angiopathy or hypertensive microangiopathy).

Regarding other imaging techniques, amyloid positron emission tomography (PET) (florbetapir or florbetaben or flutemetamol F-18) was exclusively carried out to diagnose AD when the lumbar puncture could not be performed or, in some rare cases, that CSF biomarker results were unclear [33]. F-18 fluorodeoxyglucose (FDG)-PET was performed to detect any pattern of abnormal cortical hypometabolism in suspected neurodegenerative pathologies [33], commonly in the extended FTD spectrum differential diagnosis. Dopamine transporter imaging scan (DAT-SCAN) and single-photon emission computed tomography (SPECT) were carried out for evaluation in some patients with cognitive impairment with associated parkinsonism [34,35].

The European Association of Nuclear Medicine procedure guidelines for brain PET imaging using [^18^F]FDG were followed for the acquisition protocol [36]. Subjects fasted for at least 4 h, 20–30 min before [^18^F]FDG administration, and during the uptake phase, all patients were positioned comfortably in a quiet, dimly lit room. A total scan length of 20 min image was acquired 20–30 min post-injection. The injected dose was 155 ± 25 MBq [^18^F]FDG. The glycemia levels measured before [^18^F]FDG injection were under 200 mg/dL. Amyloid PET scanning was realized with a similar protocol of attention. 

#### 2.4.4. Biomarker Data

##### Cerebrospinal Fluid Samples

**Figure 2:**
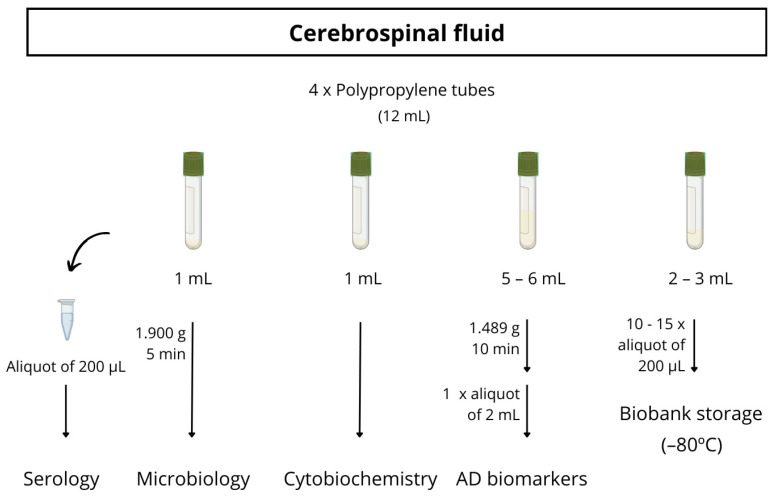
Schematic procedure for CSF sample processing.

Almost all participants underwent a lumbar puncture to obtain CSF biomarkers, generally with a 20G traumatic lumbar puncture needle. The CSF was collected between 9 and 11 a.m. in three polystyrene tubes and one polypropylene tube. The polypropylene tube is used for the detection of AD biomarkers (Aβ42, Aβ40, total tau, p-tau181, NfL, Aβ42/Aβ40, Aβ42/t-tau). The other two polystyrene tubes are used for standard cytobiochemistry analytics (macroscopic examination, erythrocytes, leukocytes, glucose, proteins) and microbiological investigations (a bacteriologic culture and treponema pallidum total antibodies). The last polystyrene tube is collected for biobank storage. The full protocol for CSF processing is detailed in Figure 2.

##### Blood and Plasma Samples

**Figure 3:**
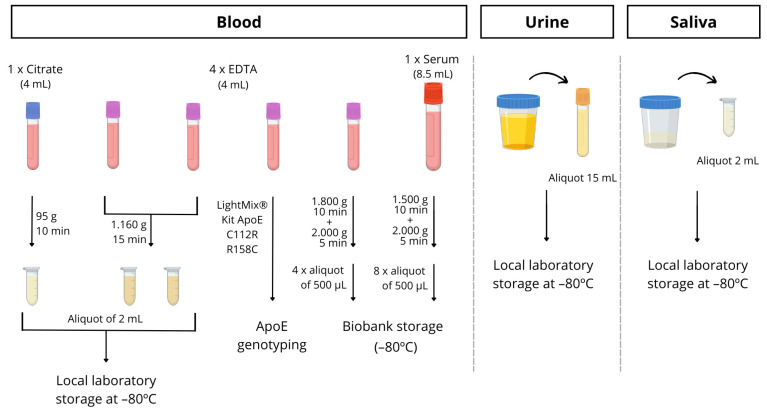
Schematic procedure for blood, urine and saliva sample processing.

Blood is drawn from all participants utilizing routine clinical practice. Specifically, 6 tubes of blood are collected, with 1 8.5 mL tube without anticoagulant for biobank storage, a 4 mL citrate tube for the detection of microRNAs in platelets, and 4 tubes of 4 mL with EDTA. Among the latter tubes, a whole blood sample was used for Apolipoprotein E genotyping, and it was determined following the manufacturer protocol by PCR using LightMix^®^ Kit ApoE C112R R158C from Roche Diagnostics (https://www.roche-as.es/lightmix_global, accessed on 23 June 2023). A specific ApoE genotype informed consent was always obtained. Another whole blood EDTA tube for biobank storage and two tubes for processing in the laboratory and plasma separation were utilized. So, these aliquots were centrifuged within 1–2 h of collection and stored at −80 °C until required for analysis. The full protocol for processing the blood is described in detail in Figure 3.

##### Other Biological Samples

In some patients, with their informed consent, urine or saliva samples were collected in a sterile bottle and immediately stored at −80 °C until analysis. 

Saliva samples were collected by spitting into sterile bottles (between 10 and 12 a.m.). Participants rinsed their mouths before saliva collection. Samples were then aliquoted into 1.5 mL tubes, and those with visible blood contamination were excluded from this study. Finally, samples were stored at −80 °C until analysis (see Figure 3).

### 2.5. Data Integration

Importantly, each participant received a unique code within the study, and all the participant’s information is integrated into the database. Specifically, sociodemographic and clinical information, genetic and neuroimaging biomarkers, and neuropsychological assessment for each participant are recorded in the database. Finally, information from these different data categories can be easily integrated for further analysis.

### 2.6. Statistical Analyses 

The analysis was performed with the IBM Statistical Package for the Social Sciences version 23.0 (SPSS, Inc., Chicago, IL, USA). For descriptive analysis, categorical variables were expressed as frequencies and percentages (%), and numerical variables were expressed as medians and interquartile ranges (IQRs). The non-parametric Kruskal–Wallis test was used to determine the differences between groups, and correlations were made using Pearson correlations. In all cases, statistical significance was set at a *p*-value < 0.05.

## 3. Results

### 3.1. Cohort Description

Table S2 shows the variables collected over the years. As can be seen, all clinical variables, neuropsychological tests, imaging techniques, most sociodemographic factors and CSF and blood samples were collected from 2017. However, saliva and urine samples were only collected from 2017 to 2019 inclusive. Also, other variables, such as CSF (Aβ40, NfL), physical activity, hand dominance, anthropometric measures, and genetics (ApoE), were gradually incorporated in recent years (see Supplementary Material).

The developed cohort now includes 1249 participants, with demographic and clinical data, blood, and CSF samples from all of them, although with fewer data on physical activity (*n* = 673), hand dominance (*n* = 348), and anthropometric measures (*n* = 373), as these variables were included later. Regarding the other variables, neuropsychological battery (*n* = 1179), biomarkers in CSF (*n* = 1097), and ApoE genotype (*n* = 900) data were available for most of the participants, while for MRI (*n* = 642) and amyloid PET (*n* = 182), data were available for less than half the participants.

The clinical classification of the participants is based on neuropsychological test scores, CSF biomarkers levels, amyloid PET results, and clinical findings by consensus. In this sense, the patients were classified into groups, as previously defined: SMC (*n* = 165), MCI-AD (*n* = 302), mild dementia AD (*n* = 215), moderate or severe dementia AD (*n* = 30), DLB (*n* = 10), FTLD (*n* = 61), and other (*n* = 466) (see Table 1). Among the AD stages, CSF Aβ42 shows statistical differences between the AD groups (*p* = 0.022). Moreover, Pearson correlations show negative correlations between CDR and Aβ42 (r = −0.156, *p* = 0.0004) and a positive correlation between MMSE and Aβ42 (r = 0.162, *p* = 0.0002) in the AD group. As expected in the ApoE genotype analysis, the groups with AD had a higher percentage of patients carrying the ε 4 allele. Regarding biomarkers in CSF, higher levels of t-tau, p-tau, and Aβ42/t-tau ratio, and lower levels of Aβ42 and Aβ42/Aβ40 ratio, were observed in the AD groups. The FTLD group had the highest levels of NfL. On the other hand, worse scores on neuropsychological tests were found in the AD groups and worsened with disease progression.

### 3.2. Relationship between ApoE Genotype and Clinical Variables

According to the ApoE genotype of the patients in our cohort (*n* = 900), they are classified from majority to minority as follows: E3/E3 (*n* = 529, 58.8%), E3/E4 (*n* = 263, 29.2%), E2/E3 (*n* = 45, 5.0%), E4/E4 (*n* = 42, 4.7%), E2/E4 (*n* = 19, 2.1%), and E2/E2 (*n* = 2, 0.2%). As expected, the majority group was patients containing the E3/E3 genotype, and the minority group included patients with the E2/E2 genotype. Therefore, it was decided to classify AD and non-AD patients into ApoE4 non-carriers, ApoE4 heterozygous, and ApoE4 homozygous in order to see differences among the groups (Table 2 and Table 3), as well as correlations between CSF biomarkers and neuropsychological tests in the different subgroups (Table 4 and Table 5). As can be seen in Table 2, among non-AD patients, there are significant differences in terms of Aβ42, p-tau, and the ratios. Nevertheless, in AD patients (Table 3), there are differences in Aβ42 among homozygous and the other groups and RBANS.DM between non-carriers and the other groups.

Regarding correlations between neuropsychology and CSF biomarkers among non-AD participants, it should be noted that the ADCS-ADL-MCI test only has significant correlations with most of the CSF biomarkers in the non-carrier group, and the MMSE and RBANS.DM tests in the non-carriers and the heterozygous group (see Table 4). On the other hand, AD participants showed other patterns, as the homozygous group had a negative correlation with t-tau, p-tau, and Aβ42/t-tau with the ADCS-ADL-MCI. And, as well as the non-AD patients, the non-carriers and the heterozygous group showed negative correlations between CSF biomarkers and the MMSE and RBANS.DM tests (see Table 5). These results are easily interpreted as the logical outcome in this type of registries.

## 4. Discussion

The Alzheimer’s Disease research group from Hospital La Fe (Valencia, Spain) developed the VALCODIS cohort with demographic, clinical, and biochemical data from more than 1000 participants (2017–2023). These participants were recruited along previous research projects focused on the identification of early and minimally invasive AD diagnosis biomarkers (e.g., metabolites, lipids, proteins, microRNAs…). The cohort participants were clinically and accurately classified according to CSF biomarker levels, neuropsychological evaluation, and neuroimaging results. Their biological samples (CSF, plasma…) obtained at diagnosis time were kept at −80 °C. This database will allow for the initiation of further transversal and longitudinal studies, involving high-viability projects. The research studies from the VALCODIS cohort will focus on improvements in clinical trial design, early and efficient treatments, personalized medicine, and the identification of new therapeutic targets. In general, all these advances could involve an important change in clinical guides.

From the demographic and clinical variables of the groups in this cohort, it can be seen that the CSF biomarkers vary across AD development, similar to neuropsychology. Traditionally, it was thought that the progression of Aβ and tau in the brain remained consistent among individuals, although recent work has shown variability in AD patients [37,38]. As expected, and as in other studies in AD cohorts [39,40], as cognitive status worsens, a decrease in Aβ42 and Aβ40 and an increase in t-tau and p-tau are observed. On the other hand, NfL has been used as a marker of neuroaxonal injury in amyotrophic lateral sclerosis, frontotemporal dementia, and multiple sclerosis, although, currently, it appears to be a marker of neurodegeneration in neurodegenerative dementias [41,42], including AD. Thus, it was corroborated that the group with higher levels of NfL was the FTLD. Also, in the AD group, an increase was observed with respect to patients with subjective memory complaints, DLB or other patients such as those with vascular dementia.

Regarding the results obtained in our cohort regarding the ApoE genotype, we can conclude that the most prevalent genotype is E3/E3. In line with other studies already published [43,44,45], most non-AD participants did not carry the E4 variant (79.6%), while in the AD participants, the E4 heterozygous was the majority (46.8%), slightly over the non-carriers. In addition, only 2.9% of the non-AD participants were E4 homozygous, and 7.8% pertained to the AD group. Interestingly, homozygous non-AD participants are more cognitively impaired than non-carriers or heterozygous, whereas homozygous AD patients do not differ as much from the other participants diagnosed with AD.

The main limitation of this cohort is the lack of all variables for all participants. In fact, some sociodemographic, anthropometric, and genetic variables were included along the years (body mass index, educational background, smoking, alcohol consumption, hand dominance, ApoE genotype), while the collection of other date was stopped (saliva, urine samples). Cohort recruitment is a dynamic process, in which the most relevant and complete information is defined over time, taking benefit from the routine clinical practice and experience. Nevertheless, many variables are included, available for all participants from 2021. In addition, the number of participants with all these relevant data is expected to increase in the following years as a VALCODIS cohort II, where other types of data relevant to the study of the disease are expected to be included. Actually, further plasma biomarker determinations are required to advance in the general screening of people at risk.

Also, in the near future, improvements are expected in the identification of other neurodegenerative pathologies with cognitive impairment but without specific biomarkers (e.g., paralysis supranuclear progressive, vascular dementia), as well as to incorporate new neuropsychological tests focused on the evaluation of patients’ behavioural impairment and caregivers burn-out syndrome. Finally, a further longitudinal study will be carried out to identify reliable prognosis biomarkers and increase our knowledge of the pathophysiological pathways involved in AD and the different AD subtypes.

## Figures and Tables

**Table 1:** Demographic and clinical participants’ description.

Variables	SMC (*n* = 165)	MCI AD (*n* = 302)	Mild Dementia AD (*n* = 215)	Moderate or Severe Dementia AD (*n* = 30)	DLB (*n* = 10)	FTLD (*n* = 61)	Other (VaD…) (*n* = 466)
Age (years, Median (IQR))	63 (59–68)	71 (67–74)	72 (68–74)	70 (65–73)	71 (69–74)	67 (62–71)	67 (61–73)
Gender (female *n* (%))	93 (56.4%)	180 (59.6%)	132 (61.4%)	23 (76.7%)	6 (60.0%)	37 (60.7%)	250 (53.6%)
ApoE (ε4 carrier *n* (%)) (*n* = 900)	19 (11.5%)	123 (40.7%)	77 (35.8%)	16 (53.3%)	1 (10.0%)	5 (8.2%)	64 (13.7%)
Aβ42 (pg·mL^−1^, median (IQR))	1224 (985–1468)	644 (496–783)	600 (453–737)	544 (452–659)	841 (791–1238)	1093 (858–1487)	1087 (809–1420)
Aβ40 (pg·mL^−1^, median (IQR)) (*n* = 592)	11,368 (9783–14,193)	13,419 (10,599–16,012)	12,572 (10,624–15,663)	10,420 (7730–12,684)	12,136 (7866–12,989)	11,727 (8641–15,968)	11,794 (8976–14,637)
t-tau (pg·mL^−1^, median (IQR))	225 (159–310)	593 (399–806)	640 (452–879)	699 (410–1008)	263 (156–388)	317 (226–519)	278 (187–394)
p-tau181 (pg·mL^−1^, median (IQR))	36 (28–47)	96 (66–134)	98 (73–141)	101 (72–149)	39 (24–58)	40 (32–61)	42 (30–58)
Aβ42/Aβ40 median (IQR) (*n* = 591)	0.108 (0.099–0.116)	0.050 (0.043–0.058)	0.049 (0.040–0.055)	0.051 (0.041–0.057)	0.102 (0.062–0.111)	0.106 (0.089–0.112)	0.107 (0.082–0.114)
Aβ42/t-tau median (IQR) (*n* = 898)	0.190 (0.150–0.240)	0.890 (0.650–1.310)	1.050 (0.710–1.645)	1.180 (0.805–1.750)	0.250 (0.163–0.467)	0.300 (0.230–0.383)	0.300 (0.230–0.383)
NfL (pg·mL^−1^, median (IQR)) (*n* = 595)	558 (442–759)	1010 (768–1359)	1157 (885–1557)	1112 (969–1560)	795 (601–949)	2029 (919–3976)	782 (512–1236)
ADCS-ADL-MCI (score, median (IQR))	47 (44–50)	43 (38–46)	36 (31–40)	26 (18–33)	39 (33–44)	31 (23–41)	42 (35–46)
CDR (score, median (IQR))	0 (0–0.5)	0.5 (0.5–0.5)	0.5 (0.5–1)	2 (2–2)	0.5 (0.5–1)	1 (0.5–1)	0.5 (0–0.5)
FAQ (score, median (IQR))	1 (0–2)	4 (1–6)	14 (11–18)	23 (18–25)	7 (3–16)	12 (7–22)	5 (1–10)
MMSE (score, median (IQR))	29 (28–30)	25 (22–27)	21 (17–24)	14 (10–18)	23 (17–25)	23 (18–25)	25 (22–28)
RBANS.DM (score, median (IQR))	98 (92–102)	56 (44–78)	48 (40–56)	40 (40–48)	75 (42–83)	56 (44–78)	68 (52–84)
GDS (score, median (IQR))	9 (5–16)	8 (5–13)	10 (6–16)	9 (6–14)	18 (8–23)	13 (6–18)	12 (6–19)

ADCS-ADL-MCI: the Alzheimer’s Disease Cooperative Study–Activities of Daily Living Inventory-Mild Cognitive Impairment; CDR: Clinical Dementia Rating; FAQ: Mini-Cog-Functional Activities Questionnaire; GDS: Geriatric Depression Scale; MMSE: Mini-mental State Examination; NfL: Neurofilament light chain; RBANS: Repeatable battery for the Assessment of Neuropsychological Status; SMC: Subjective memory complaint; VaD: Vascular Dementia.

**Table 2:** Clinical non-AD participants’ description concerning ApoE genotype.

Variables	Non-Carrier Participants (*n* = 378, 79.6%)	ε4 Heterozygous Participants (*n* = 83, 17.5%)	ε4 Homozygous Participants (*n* = 14, 2.9%)	*p*-Value ^a^
Aβ42 (pg·mL^−1^, median (IQR))	1196 (925–1544)	901 (673–1242)	515 (370–1158)	<0.001
Aβ40 (pg·mL^−1^, median (IQR))	11,727 (9187–14,883)	12,232 (8806–14,168)	10,637 (7325–15,474)	0.695
t-tau (pg·mL^−1^, median (IQR))	259 (182–361)	269 (172–444)	409 (236–652)	0.051
p-tau181 (pg·mL^−1^, median (IQR))	36 (27–49)	42 (31–61)	71 (36–96)	0.002
Aβ42/Aβ40 median (IQR)	0.109 (0.100–0.116)	0.087 (0.069–0.105)	0.053 (0.035–0.102)	<0.001
Aβ42/t-tau median (IQR)	0.210 (0.170–0.290)	0.285 (0.180–0.460)	0.900 (0.270–1.505)	<0.001
NfL (pg·mL^−1^, median (IQR))	760 (516–1205)	694 (440–1074)	899 (480–2395)	0.151
ADCS-ADL-MCI (score, median (IQR))	42 (35–47)	44 (37–48)	43 (37–47)	0.203
CDR (score, median (IQR))	0.5 (0.5–0.5)	0.5 (0.5–0.5)	0.5 (0.5–0.5)	0.534
FAQ (score, median (IQR))	4 (1–10)	3 (0–7)	1 (0–9)	0.135
MMSE (score, median (IQR))	26 (23–28)	27 (24–29)	24 (22–27)	0.174
RBANS.DM (score, median (IQR))	78 (56–95)	78 (60–94)	56 (40–92)	0.288

^a^ Kruskal-Wallis Test.

**Table 3:** Clinical AD participants’ description concerning ApoE genotype.

Variables	Non-Carrier Participants (*n* = 193, 45.4%)	ε4 Heterozygous Participants (*n* = 199, 46.8%)	ε4 Homozygous Participants (*n* = 33, 7.8%)	*p*-Value ^a^
Aβ42 (pg·mL^−1^, median (IQR))	643 (482–796)	632 (498–752)	501 (390–606)	<0.001
Aβ40 (pg·mL^−1^, median (IQR))	12,907 (10,349–16,115)	13,145 (10,751–15,752)	11,049 (9419–13,656)	0.073
t-tau (pg·mL^−1^, median (IQR))	623 (443–859)	626 (403–867)	587 (368–927)	0.854
p-tau181 (pg·mL^−1^, median (IQR))	103 (71–141)	104 (70–143)	86 (69–131)	0.738
Aβ42/Aβ40 median (IQR))	0.050 (0.042–0.059)	0.050 (0.043–0.056)	0.045 (0.036–0.055)	0.098
Aβ42/t-tau median (IQR))	0.930 (0.660–1.375)	0.990 (0.685–1.420)	1130 (0.700–1.685)	0.290
NfL (pg·mL^−1^, median (IQR))	1108 (882–1556)	1051 (811–1409)	901 (724–1242)	0.084
ADCS-ADL-MCI (score, median (IQR))	39 (33–45)	39 (34–45)	38 (33–42)	0.493
CDR (score, median (IQR))	0.5 (0.5–1)	0.5 (0.5–1)	0.5 (0.5–1)	0.226
FAQ (score, median (IQR))	7 (3–14)	7 (2–13)	8 (5–14)	0.878
MMSE (score, median (IQR))	22 (19–26)	23 (19–26)	24 (18–26)	0.946
RBANS.DM (score, median (IQR))	56 (44–71)	48 (40–64)	44 (40–56)	0.001

^a^ Kruskal-Wallis Test.

**Table 4:** Correlations between CSF biomarkers and neuropsychological tests among clinical non-AD participants’ concerning their ApoE genotype.

	r ^a^ (*p* Value)	ADCS-ADL-MCI	CDR	FAQ	MMSE	RBANS.DM
non-carrier	Aβ42	0.062 (0.262)	−0.020 (0.713)	−0.050 (0.364)	0.066 (0.228)	0.078 (0.163)
	Aβ40	0.061 (0.294)	−0.043 (0.461)	−0.074 (0.209)	0.015 (0.790)	0.035 (0.555)
	t-tau	−0.199 (<0.001)	0.171 (0.002)	0.199 (<0.001)	−0.254 (<0.001)	−0.144 (0.010)
	p-tau	−0.139 (0.012)	0.100 (0.069)	0.139 (0.012)	−0.256 (<0.001)	−0.174 (0.002)
	Aβ42/Aβ40	0.040 (0.496)	−0.006 (0.924)	−0.063 (0.282)	0.132 (0.022)	0.103 (0.083)
	Aβ42/t-tau	−0.202 (<0.001)	0.144 (0.010)	0.202 (<0.001)	−0.288 (<0.001)	−0.196 (0.001)
	NfL	−0.318 (<0.001)	0.313 (<0.001)	0.350 (<0.001)	−0.285 (<0.001)	−0.287 (<0.001)
ε4 heterozygous	Aβ42	0.224 (0.070)	−0.279 (0.024)	−0.213 (0.093)	0.334 (0.005)	0.191 (0.119)
	Aβ40	0.067 (0.606)	−0.053 (0.684)	−0.059 (0.658)	0.025 (0.841)	−0.019 (0.884)
	t-tau	−0.046 (0.713)	0.170 (0.177)	0.152 (0.234)	−0.415 (<0.001)	−0.313 (0.009)
	p-tau	−0.140 (0.261)	0.207 (0.098)	0.182 (0.153)	−0.440 (<0.001)	−0.381 (0.001)
	Aβ42/Aβ40	0.255 (0.045)	−0.425 (0.001)	−0.274 (0.036)	0.458 (<0.001)	0.252 (0.045)
	Aβ42/t-tau	−0.255 (0.046)	0.372 (0.003)	0.320 (0.013)	−0.579 (<0.001)	−0.372 (0.002)
	NfL	−0.336 (0.008)	0.356 (0.005)	0.367 (0.004)	−0.419 (0.001)	−0.255 (0.042)
ε4 homozygous	Aβ42	0.251 (0.588)	−0.345 (0.449)	−0.626 (0.374)	0.439 (0.325)	0.718 (0.108)
	Aβ40	0.234 (0.614)	−0.445 (0.316)	−0.448 (0.552)	0.296 (0.519)	0.307 (0.555)
	t-tau	−0.117 (0.802)	0.012 (0.979)	−0.019 (0.981)	−0.263 (0.569)	−0.126 (0.812)
	p-tau	−0.138 (0.768)	−0.079 (0.867)	0.436 (0.564)	−0.357 (0.431)	−0.602 (0.206)
	Aβ42/Aβ40	0.347 (0.445)	−0.242 (0.601)	−0.929 (0.071)	0.607 (0.149)	0.888 (0.018)
	Aβ42/t-tau	−0.370 (0.414)	0.465 (0.294)	0.999 (0.001)	−0.706 (0.076)	−0.754 (0.084)
	NfL	−0.676 (0.096)	0.854 (0.014)	−0.252 (0.748)	−0.786 (0.036)	−0.453 (0.368)

^a^ Pearson correlation coefficient (r).

**Table 5:** Correlations between CSF biomarkers and neuropsychological tests among clinical AD participants’ concerning their ApoE genotype.

	r ^a^ (*p* Value)	ADCS-ADL-MCI	CDR	FAQ	MMSE	RBANS.DM
non-carrier	Aβ42	0.201 (0.007)	−0.232 (0.001)	−0.226 (0.002)	0.243 (0.001)	0.315 (<0.001)
	Aβ40	0.158 (0.049)	−0.201 (0.012)	−0.149 (0.065)	0.109 (0.173)	0.061 (0.458)
	t-tau	0.037 (0.624)	0.066 (0.371)	0.073 (0.322)	−0.253 (0.001)	−0.306 (<0.001)
	p-tau	0.008 (0.919)	0.071 (0.339)	0.093 (0.211)	−0.229 (0.002)	−0.334 (<0.001)
	Aβ42/Aβ40	0.069 (0.396)	−0.036 (0.653)	−0.102 (0.205)	0.166 (0.037)	0.333 (<0.001)
	Aβ42/t-tau	−0.015 (0.846)	0.127 (0.085)	0.148 (0.046)	−0.293 (<0.001)	−0.349 (<0.001)
	NfL	−0.170 (0.035)	0.116 (0.150)	0.206 (0.010)	−0.138 (0.087)	−0.136 (0.098)
ε4 heterozygous	Aβ42	0.103 (0.169)	−0.099 (0.177)	−0.084 (0.253)	0.106 (0.148)	0.109 (0.145)
	Aβ40	0.044 (0.596)	−0.168 (0.043)	−0.066 (0.427)	0.032 (0.702)	−0.068 (0.426)
	t-tau	−0.108 (0.150)	0.132 (0.071)	0.120 (0.101)	−0.192 (0.008)	−0.297 (<0.001)
	p-tau	−0.086 (0.250)	0.053 (0.466)	0.055 (0.452)	−0.130 (0.073)	−0.260 (<0.001)
	Aβ42/Aβ40	0.061 (0.470)	0.016 (0.846)	−0.036 (0.670)	0.096 (0.250)	0.188 (0.027)
	Aβ42/t-tau	−0.108 (0.151)	0.117 (0.110)	0.094 (0.197)	−0.162 (0.026)	−0.253 (0.001)
	NfL	−0.188 (0.023)	0.123 (0.139)	0.199 (0.016)	−0.118 (0.154)	−0.303 (<0.001)
ε4 homozygous	Aβ42	0.265 (0.174)	−0.272 (0.146)	−0.127 (0.504)	0.163 (0.389)	−0.018 (0.925)
	Aβ40	−0.022 (0.918)	−0.030 (0.889)	0.175 (0.414)	−0.092 (0.670)	−0.011 (0.960)
	t-tau	−0.537 (0.003)	0.356 (0.054)	0.214 (0.255)	−0.283 (0.130)	−0.196 (0.307)
	p-tau	−0.426 (0.024)	0.390 (0.033)	0.294 (0.115)	−0.369 (0.045)	−0.205 (0.286)
	Aβ42/Aβ40	0.273 (0.197)	−0.272 (0.198)	−0.424 (0.039)	0.245 (0.249)	−0.050 (0.819)
	Aβ42/t-tau	−0.591 (0.001)	0.396 (0.030)	0.215 (0.255)	−0.249 (0.185)	−0.149 (0.440)
	NfL	−0.168 (0.433)	0.257 (0.226)	0.184 (0.389)	−0.009 (0.965)	−0.078 (0.724)

^a^ Pearson correlation coefficient (r).

## Data Availability

The data that support the findings of this study are available from the corresponding author upon a reasonable request.

## Supplementary Materials

The following supporting information can be downloaded at: https://www.mdpi.com/article/10.3390/jcm13164735/s1, Table S1: The VALCODIS cohort classification criteria; Table S2: Cohort data collection over the years.
