# Supplementary material for: Insights from a 7-Year Dementia Cohort (VALCODIS): ApoE Genotype Evaluation
Source: J Clin Med. 2024 Aug 12;13(16):4735. doi: 10.3390/jcm13164735 (PMC11355866; doi:10.3390/jcm13164735)
Supplement: Supplementary file 1 [file jcm-13-04735-s001.zip › jcm-3136154-supplementary.pdf]

Table S1. The VALCODIS cohort classification criteria.

**Participants Group**	**Classification Criteria**
Subjective memory complaint	● negative levels for CSF AD biomarkers (β-amyloid-42 > 725 pg·mL−1, t-tau < 485 pg·mL−1, p-tau181 < 56 pg·mL−1) or negative PET amyloid.<br>● normal cognitive tests (ADCS-ADL-MCI > 44, CDR = 0 to 0.5, FAQ < 9, MMSE between 27-30, RBANS.DM ≥ 85).<br>● Do not fulfil the criteria for MCI or any dementia.
Mild cognitive impairment AD	● positive CSF AD biomarkers (β-amyloid-42 < 725 pg·mL−1, t-tau > 485 pg·mL−1, p-tau181 > 56 pg·mL−1) or positive PET amyloid.<br>● mild cognitive impairment (ADCS-ADL-MCI < 44, CDR ≤ 0.5, FAQ < 9, MMSE of 24 – 30, RBANS.DM ≤ 85).
Mild Dementia AD	● positive CSF AD biomarkers (β-amyloid-42 < 725 pg·mL−1, t-tau > 485 pg·mL−1, p-tau181 > 56 pg·mL−1) or positive PET amyloid.<br>● cognitive impairment (ADCS-ADL-MCI < 44, CDR = 1, FAQ > 9, MMSE ≤ 27, RBANS.DM ≤ 85)
Moderate and severe dementia AD	● positive CSF AD biomarkers (β-amyloid-42 < 725 pg·mL−1, t-tau > 485 pg·mL−1, p-tau181 > 56 pg·mL−1) or positive PET amyloid.<br>● cognitive impairment (ADCS-ADL-MCI < 44, CDR = 2 - 3, FAQ > 9, MMSE <21, RBANS.DM ≤ 85)
DLB	● negative levels for CSF AD biomarkers (β-amyloid-42 > 725 pg·mL−1, t-tau < 485 pg·mL−1, p-tau181 < 56 pg·mL−1)<br>● Core criteria of DLB:<br>○ Fluctuating cognition with pronounced variations in attention and alertness<br>○ Recurrent visual hallucinations<br>○ REM sleep behaviour disorder<br>○ One or more spontaneous cardinal features of parkinsonism (bradykinesia, rest tremor, rigidity)<br>● Supportive clinical features:<br>○ Severe sensitivity to antipsychotic agents<br>○ Postural instability<br>○ Repeated falls<br>○ Syncope or other transient episodes of unresponsiveness<br>○ Severe autonomic dysfunction (e.g., constipation, orthostatic hypotension, urinary incontinence)<br>○ Hypersomnia<br>○ Hyposmia<br>○ Hallucinations in other modalities<br>○ Systematised delusions<br>○ Apathy, anxiety, and depression
FTLD	● negative levels for CSF AD biomarkers (β-amyloid-42 > 725 pg·mL−1, t-tau < 485 pg·mL−1, p-tau181 < 56 pg·mL−1)<br>● Three of six clinical feature for possible DLFTs:<br>○ Disinhibition<br>○ Apathy/inertia<br>○ Loss of sympathy/empathy<br>○ Perseverative/compulsive behaviours<br>○ Hyperorality<br>○ Dysexecutive neuropsychological profile<br>● Probable FTD requires the same clinical criteria, + functional decline and imaging findings (i.e., frontal and/or temporal lobe atrophy, hypometabolism, or hypoperfusion)<br>● Definite FTLD pathology with evidence of known pathogenic mutation.<br>● For PPA:

	○ Most prominent clinical feature is difficulty with language<br>○ These deficits are the principal cause of impaired daily living activities<br>○ Aphasia should be the most prominent deficit at symptom onset and for the initial phases of the disease<br>○ Could be supported by neuroimaging findings (atrophy, hypoperfusion, hypometabolism), or confirmed by genetic data
Other	● Patients with or without dementia but with negative biomarkers for AD, psychiatric patients, vascular dementia, or patients unable to be classified into previous groups, without CSF biomarkers/amyloid PET or without neuropsychological evaluation.

ADCS-ADL-MCI: the Alzheimer's Disease Cooperative Study–Activities of Daily Living Inventory-Mild Cognitive Impairment; CDR: Clinical Dementia Rating; CSF: cerebrospinal fluid; DLB: dementia with Lewy bodies; FAQ: Mini-Cog-Functional Activities Questionnaire; FTLD: frontotemporal lobar degeneration; MMSE: Mini-mental State Examination; RBANS: Repeatable battery for the Assessment of Neuropsychological Status.

Table S2. Cohort data collection over the years

**Variables**		**2017**	**2018**	**2019**	**2020**	**2021**	**2022**	**2023**
**Sociodemographic**	Date of birth, age, gender, and educational level	X	X	X	X	X	X	X
	Employment and professional status	X	X	X	X	X	X	X
	Tobacco and alcohol use	X	X	X	X	X	X	X
	Physical activity			X	X	X	X	X
	Hand dominance						X	X
**Anthropometric**	Weight, height and BMI					X	X	X
**Clinical**	Pharmacological profile (statins, fibrates, antidepressants, antiepileptics, anticoagulants, anti-inflammatories…)	X	X	X	X	X	X	X
	Symptoms (memory, language, behaviour...l)	X	X	X	X	X	X	X
	Comorbidities (dyslipidemia, diabetes, hypertension, heart disease…)	X	X	X	X	X	X	X
**Genetic**	APOE		X	X	X	X	X	X
**Neuropsychological assessment**	CDR, MMSE, RBANS, FAQ, ADCS-ADL-MCI and GDS	X	X	X	X	X	X	X
**Imaging techniques**	CT or MRI or PET or FDG-PET or DAT-SCAN	X	X	X	X	X	X	X
**Laboratory**	CSF	X	X	X	X	X	X	X
	Blood	X	X	X	X	X	X	X

	Urine	X	X	X				
	Saliva	X	X	X				
**CSF biomarkers**	Aβ42, t-tau, p-tau181	X	X	X	X	X	X	X
	Aβ40, Aβ42/ Aβ40, Aβ42/t-tau			X	X	X	X	X
	NfL			X	X	X	X	X

ADCS-ADL-MCI: the Alzheimer's Disease Cooperative Study–Activities of Daily Living Inventory-Mild Cognitive Impairment; BMI: Body Mass Index; CDR: Clinical Dementia Rating; CSF: cerebrospinal fluid ; CT: Computed Tomography; DAT-SCAN: Dopamine Transporter imaging Scan; FAQ: Mini-Cog-Functional Activities Questionnaire; ; FDG-PET F-18 fluorodeoxyglucose Positron Emission Tomography; GDS: Geriatric Depression Scale; MMSE: Mini-mental State Examination; MRI: Magnetic Resonance Imaging; NfL: Neurofilament light chain; RBANS: Repeatable battery for the Assessment of Neuropsychological Status; PET: Positron Emission Tomography.
